# Ankle fractures involving the posterior malleolus: patient characteristics and 7-year results in 100 cases

**DOI:** 10.1007/s00402-021-03875-3

**Published:** 2021-04-09

**Authors:** Annika Pauline Neumann, Stefan Rammelt

**Affiliations:** University Center of Orthopaedics, Trauma and Plastic Surgery, University Hospital Carl Gustav Carus at TU Dresden, Fetscherstrasse 74, 01307 Dresden, Germany

**Keywords:** Ankle, Malleolar fracture, Posterior tibia, Internal fixation, Outcome

## Abstract

**Introduction:**

The presence of a posterior malleolar (PM) fragment has a negative prognostic impact in ankle fractures. The best treatment is still subject to debate. The aim of this study was to assess the medium-to-long-term clinical and functional outcome of ankle fractures with a PM fragment in a larger patient population.

**Materials and methods:**

One hundred patients (69 women, 31 men, average age 60 years) with ankle fractures including the PM were evaluated clinically and radiographically. Patients with Bartoníček–Rammelt type 3 and 4 fracture displayed a significant female preponderance. Fixation of the PM was performed in 63% and tailored to the individual fracture pattern.

**Results:**

Internal fixation of the PM fragment was negatively correlated with the need for syndesmotic screw placement at the time of surgery (*p* = 0.010). At an average follow-up of 7.0 years, the mean Foot Function Index (FFI) was 16.5 (*SD: 21.5*), the Olerud Molander Ankle Score (OMAS) averaged 80.2 (*SD: 24*) and the American Orthopedic Foot & Ankle Society (AOFAS) ankle/hindfoot score averaged 87.5 (*SD: 19.1*). The maximum score of 100 was achieved by 44% of patients. The physical (PCS) and mental health component summary (MCS) scores of the SF-36 averaged 47.7 (*SD: 12.51*) and 50.5 (*SD: 9.36*), respectively. Range of motion was within 3.4 (*SD: 6.63*) degrees of the uninjured side. The size of the PM fragment had no prognostic value. There was a trend to lower outcome scores with slight anterior or posterior shift of the distal fibula within the tibial incisura. Patients who underwent primary internal fixation had significantly superior SF-36 MCS than patients who underwent staged internal fixation (*p* = 0.031).

**Conclusions:**

With an individualized treatment protocol, tailored to the CT-based assessment of PM fractures, favorable medium and long-term results can be expected.

## Introduction

Fractures of posterior malleolus (PM), the posterior edge of the distal tibia, occur in up to 50% of malleolar fractures [1–4]. Traditionally, the presence of a PM fragment has been associated with a less favorable prognosis in ankle fractures [1, 5–9]. It may be speculated that failure to adequately recognize and treat PM fractures in the past has contributed to less-than-optimal treatment results after ankle fractures.

Despite an increasing body of literature, the best treatment of PM fractures is still subject to debate [10–12]. For several decades, criteria for surgical fixation of a PM fragment were fragment size of 1/4 to 1/3 of the articular surface and displacement of more than 2 mm on the lateral radiograph [13–16]. With a more generous use of CT imaging and increased knowledge of the three-dimensional pathoanatomy of PM fractures, besides size and displacement, involvement of the incisura, the presence of intercalary fragments, plafond impaction, and syndesmotic instability are increasingly considered for decision making [10, 11, 17–21]. Therefore, treatment has to be tailored to the individual three-dimensional fracture pattern. Over the recent years, the goals of operative fixation have been reformulated as follows: (1) restoration of articular congruity at the distal tibia and posterior containment of the talus, (2) bone-to-bone fixation of the posterior tibiofibular ligament, and (3) restoration of the fibular notch thus facilitating reduction of the distal fibula [10, 11, 22].

The aim of our retrospective study was to assess the medium-to-long-term functional and radiographic outcome of ankle fractures with a PM fragment treated with an individualized approach in a larger patient population.

## Materials and methods

### Patient characteristics

In a retrospective chart review, we identified all patients treated surgically at our institution between January 2003 and December 2015 for ankle fractures involving the posterior malleolus. The minimum follow-up was set at 2 years. We excluded patients aged under 18 years, patients with tibial pilon fractures, concomitant fractures of the same limb, polytrauma and inability to complete the questionnaire. 281 patients who met the inclusion criteria were contacted by mail or telephone and invited for follow-up. At that time, 5 patients were deceased, 43 lived in a distant location, 44 moved to an unknown location and could not be contacted, and 89 declined to participate. The study protocol was approved by the institutional review board (ethics committee).

This left 100 patients (69 women, 31 men) who were seen for clinical follow-up between August 2018 and February 2019. All following numbers and percentages pertain to this patient cohort, unless stated otherwise. In particular, the age group over 70 years was dominated by women (85.7%). The mean age of the patients at time of fracture was 60.0 years (range, 20–83 years). Men had a mean age of 53.5 years (21–74 years) and women had a mean age of 63.0 years (range, 20–83 years) at the time of injury.

**Fig. 1:**
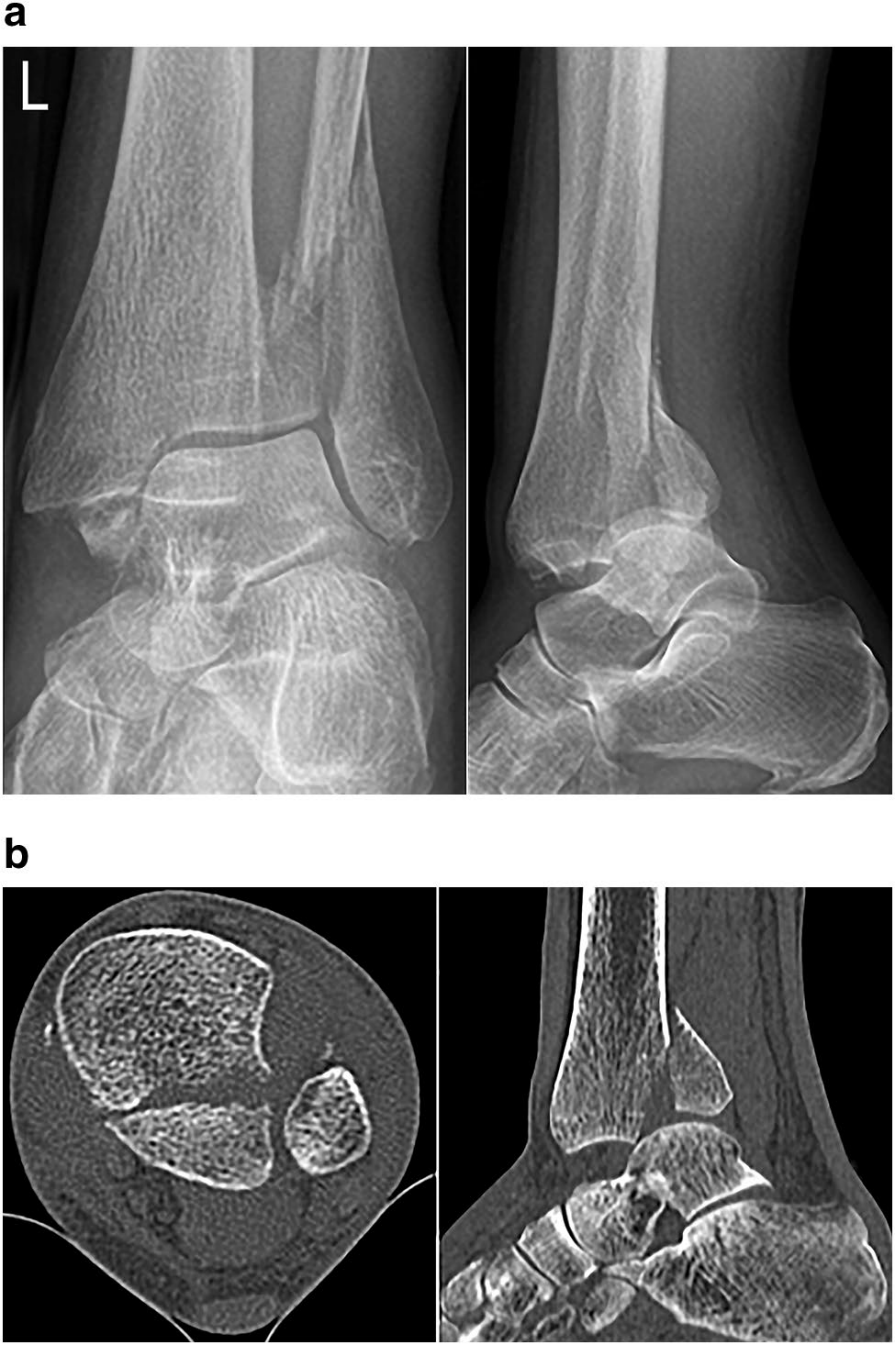
**a** Preoperative anteroposterior and lateral radiographs of a 74-year-old female patient with a quadrimalleolar fracture-dislocation of her left ankle. **b** Preoperative axial and sagittal CT images reveal a large triangular posterior malleolar fragment (Bartoníček–Rammelt type 4) and a chip-like avulsion from the anterior tibial tubercle

Diagnosis was established with radiographs in all patients and additional CT imaging in 55 cases. Of 100 ankle fractures with a PM fragment, 12% were bimalleolar fractures, 57% were trimalleolar fractures (Fig. 1) and 31% were quadrimalleolar fractures [23]: 13% trimalleolar fractures with an additional tubercule de Tillaux–Chaput fragment, 16% trimalleolar fractures with an additional Wagstaffe–LeFort fragment and 2% trimalleolar fractures with both a tubercule de Tillaux–Chaput and Wagstaffe–LeFort fragment. The right ankle was affected in 49% and the left ankle in 51%.

A Weber type B fracture occurred in 76% and a Weber type C fracture in 24%. According to the Lauge–Hansen classification [24], 46% had a pronation abduction stage 3 (PA-3) fracture, 24% a pronation external rotation stage 4 (PE-4) fracture, 4% a supination external rotation stage 3 (SE-3) fracture and 26% a SE-4 fracture. No Weber type A or supination adduction fractures were seen in this series.

Posterior malleolar fractures were classified according to Bartoníček and Rammelt [20], because this classification, in contrast to others, represents a continuum of increasing severity of injury [20, 25], is related to the mechanism of injury [26], can be used as a guide for treatment [10, 22, 27] and has been shown to be of prognostic relevance [28]. According to the Bartoníček–Rammelt classification [20], type 2 and 3 PM fractures were the most common, with 35% each. Type 1 was seen in 7% and type 4 in 23%. Intercalary fragments were found in 33 cases, most often in Bartoníček–Rammelt type 3 (*n* = 24, 72.7%), less frequently in type 2 (*n* = 7, 21.2%) and type 4 (*n* = 2, 6.1%). No intercalary fragments were seen in type 1 PM fractures.

Patients with type 1 PM fractures displayed an almost equal gender distribution (4 men vs. 3 women), while type 3 (9 men vs. 26 women) or type 4 (4 men vs. 19 women) displayed a significant female preponderance (*p* = 0.017). Patients with a Weber C, SE 3 or Bartoníček–Rammelt type 4 fracture were younger, while patients with PA 3 and Bartoníček–Rammelt type 1 fractures showed the highest patient age.

Four patients (4%) suffered from open fractures. One patient developed a compartment syndrome of the lower leg. A fasciotomy and secondary wound closure were performed. The following relevant comorbidities were noted: arterial hypertension (54%), diabetes mellitus (17%), osteoporosis (17%), peripheral arterial occlusive disease (2%), and rheumatoid arthritis (2%).

### Surgical treatment

Primary open reduction and internal fixation was performed in 53 patients. In 47 patients, primary closed reduction and external fixation was followed by staged internal fixation after soft tissue consolidation.

**Fig. 2:**
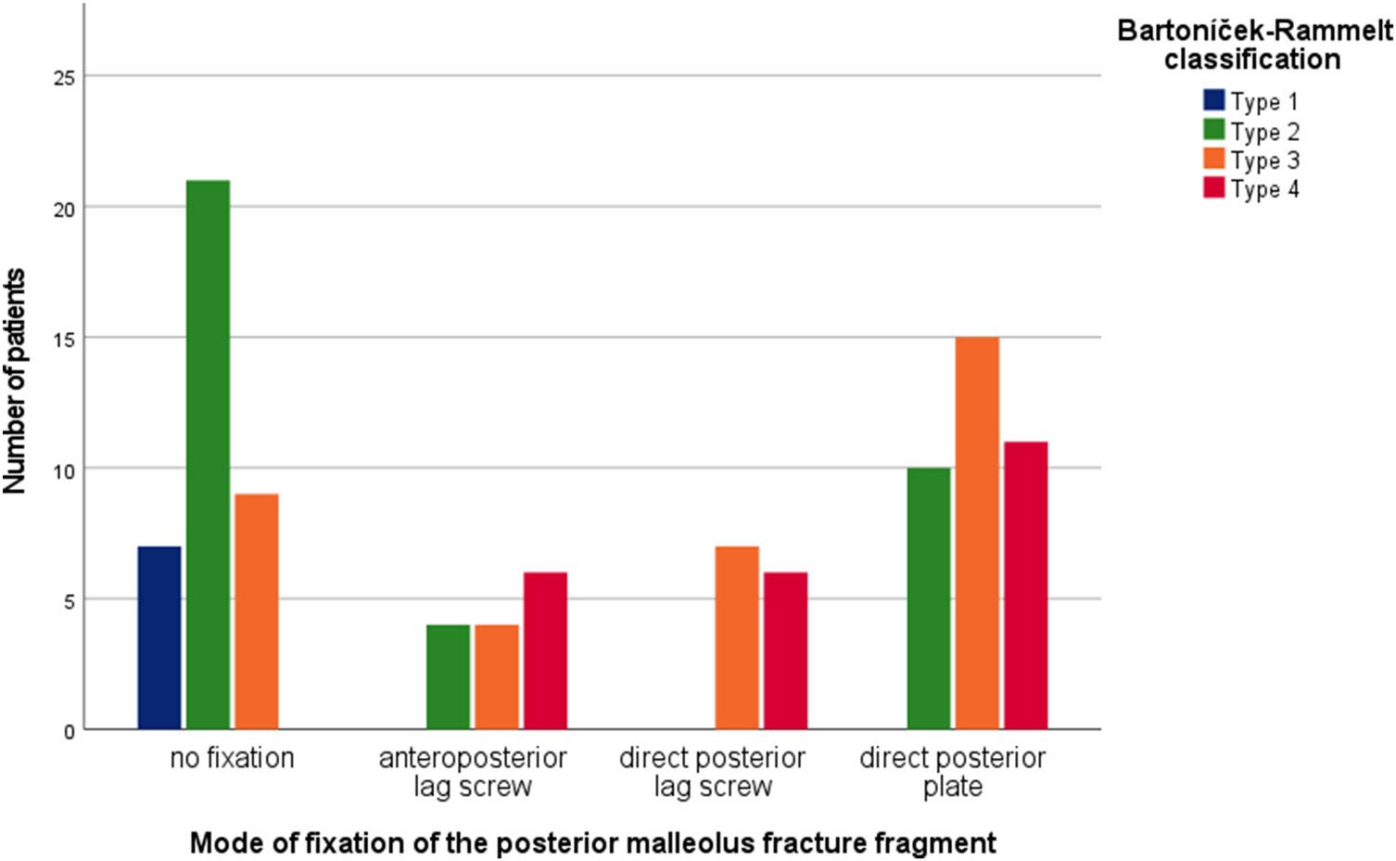
Type of posterior malleolar fracture fixation with respect to the pathoanatomy (Bartoníček–Rammelt classification [20])

**Fig. 3:**
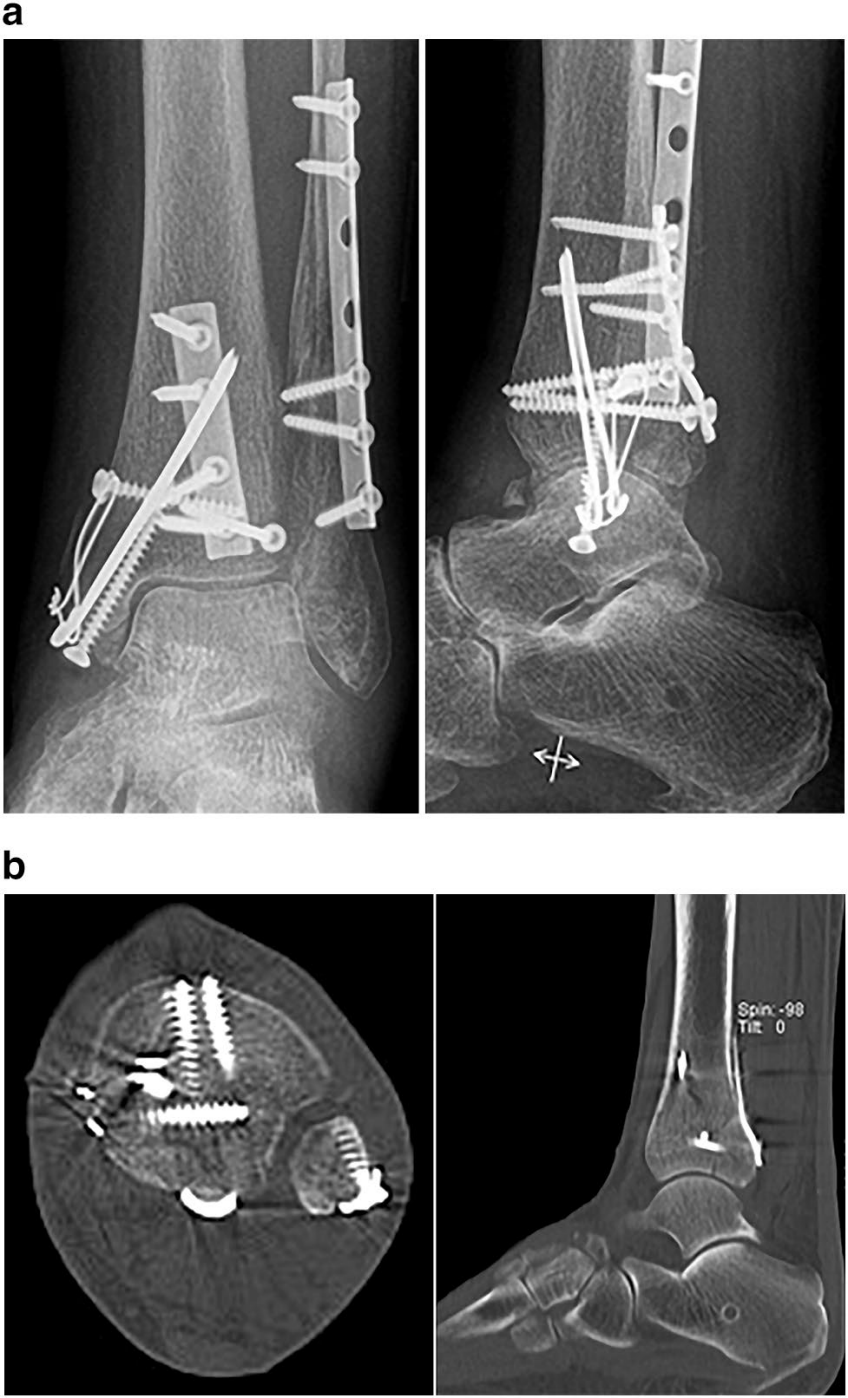
**a** Postoperative anteroposterior and lateral radiographs and **b** axial and sagittal CT scans after open reduction and posterior antiglide plate fixation of the distal tibia and fibula via a posterolateral approach and medial malleolar fixation via a medial approach all with the patient in prone position (same patient as in Fig. 1)

The PM fracture was fixed in 63%. Indirect anterior-to-posterior lag screw fixation through an anterior approach was performed in 14%. Direct fixation via posterior approaches was performed in 49% (Fig. 2). Of these, 36 PM fractures (73.5%) were fixed with a dorsal antiglide plate and 13 PM fractures (26.5%) were fixed with posterior-to-anterior lag screws. A posterolateral approach was used in 42 cases and a posteromedial approach in 7 cases. The type of internal fixation according to the fracture morphology is summarized in Fig. 3.

Of a total of 100 fibular fractures, 94 were treated by plate fixation. In one patient a fibular nail was used and in 5 cases no fibular fixation was performed. Three of these 5 cases were Maisonneuve fractures that were treated with 2 syndesmotic positioning screws. In 2 cases, the fibular fracture was deemed stable after fixation of the PM fracture.

Of 88 medial malleolar fractures, 65 (73.9%) fractures were fixed with two cancellous bone screws, 19 (21.6%) with tension band wiring, and 3 (3.4%) with a medial plate. In one case (1.1%), the medial malleolus was non-displaced and did not require fixation. The Wagstaffe and Chaput fragments were fixed with a 2.7 mm titanium screw with washer in 19 cases and with a PDS suture in 10 cases.

After fixation of all bony components of the injury, an additional syndesmotic positioning screw was placed in 15% following a positive hook test [15]. Syndesmotic positioning screws were used significantly more frequently in PE 4, Weber type C and Bartoníček–Rammelt type 2 fractures (*p* < 0.05).

### Clinical assessment

In the clinical examination at follow-up, the gait pattern, the movement sequence, the single-leg stand, tiptoe and heel gait were assessed. Range of motion at the ankle, subtalar and mid-tarsal joints were measured on both sides using a goniometer. In addition, foot deformities, skin alterations, edema, and neurovascular deficits were recorded. Clinical assessment was done by the first author (APN), who was not involved in patient treatment.

Four questionnaires were used to assess the treatment outcome: Olerud–Moleander–Ankle Score (OMAS), American Orthopaedic Foot and Ankle Society (AOFAS) Ankle/Hindfoot Scale, Foot Function Index (FFI) and Short Form Health 36 (SF-36). The scales of the OMAS and AOFAS scores range from 0 (worst result) to 100 points (best possible result). The Foot Function Index (FFI) covers the three domains foot pain, disability, and limitation of activities. It contains 23 questions that are scored on a scale from 0 (no pain/problems) to 10 (worst pain/worst problems). The best overall score is calculated as a percentage ranging from 0 as the best to 100 as the worst possible score. The German version of the FFI has been validated [29]. The Short Form Health 36 (SF-36) is a globally established, validated general health questionnaire. With a total of 36 questions, the patient's state of health is surveyed and evaluated via 8 sub-items. The results of the physical health component summary score and the mental health component summary score are compared with the results in the German norm population (mean value 50, status 1998) [30].

### Radiographic assessment

In accordance with the study protocol, no routine radiographs were obtained at follow-up. Mortise and lateral weight-bearing radiographs of both ankles were taken only if the patients had residual complaints or questions regarding implant removal. Images were assessed with regard to the Weber indices for malleolar fracture reduction (fibular length, Menard Shenton line of the ankle, medial, superior and tibiofibular clear space), residual step-offs in the joint surface and signs of posttraumatic arthritis. CT scans, if available, were used to assess the position of the distal fibula within the tibial incisura. Preoperative CT scans were available in 55% of cases. The grade of osteoarthritis on both ankles was determined using the Kellgren and Lawrence scale [31].

### Statistical workup

Statistical analysis was performed with the statistics program SPSS for Windows Version 26 (SPSS Inc., Chicago, Illinois, USA). The mean values, standard deviations, minimum, maximum, median and frequencies were calculated for the collected data. Three main criteria were defined to assess the outcome: (1) The results in the four scores, (2) side-to-side differences in the grades of osteoarthritis and (3) measured side-to-side differences in range of motion. Significance was calculated using the Chi-square test, the Mann–Whitney *U* test and the Kruskal–Wallis test for non-parametric data. The significance level was set at *p* < 0.05.

## Results

### Clinical outcome

One hundred patients were evaluated clinically at a mean follow-up of 7.0 years (83.6 months, range, 33–171 months). At the time of follow-up, 33% of patients still reported some residual pain, 29% a sensitivity to weather, and 42% a tendency of the ankle to swell upon exercise. Local hypoesthesia or dysesthesia was reported by 10% of patients. At the time of follow-up, two ankle fusions had been performed for symptomatic arthritis following deep infection.

The mean FFI at the time of follow-up was 16.5 (range, 89.5–0; SD: 21.5). The best possible result of 0 was achieved by 26% of patients. The OMAS score averaged 80.2 (range 20–100; SD: 24). The best possible result of 100 was achieved by 27% of patients. The lowest score of 20 was seen in 4% of patients. With the AOFAS ankle/hindfoot scale, the average score was 87.5 (range, 12–100; SD: 19.1). The maximum score of 100 points was achieved by 44% of patients.

The physical health component summary score (PCS) of the SF-36 averaged 47.7 (range, 19.5 to 63.6; SD: 12.51). This was slightly lower than the German population norm with a mean value of 50. In contrast, the mean value of the mental health component summary score (MCS) of the SF-36 was 50.5 (range, 24.0 to 65.1; SD: 9.36) which was slightly higher than the cumulative scale of 50 of the German norm population.

**Fig. 4:**
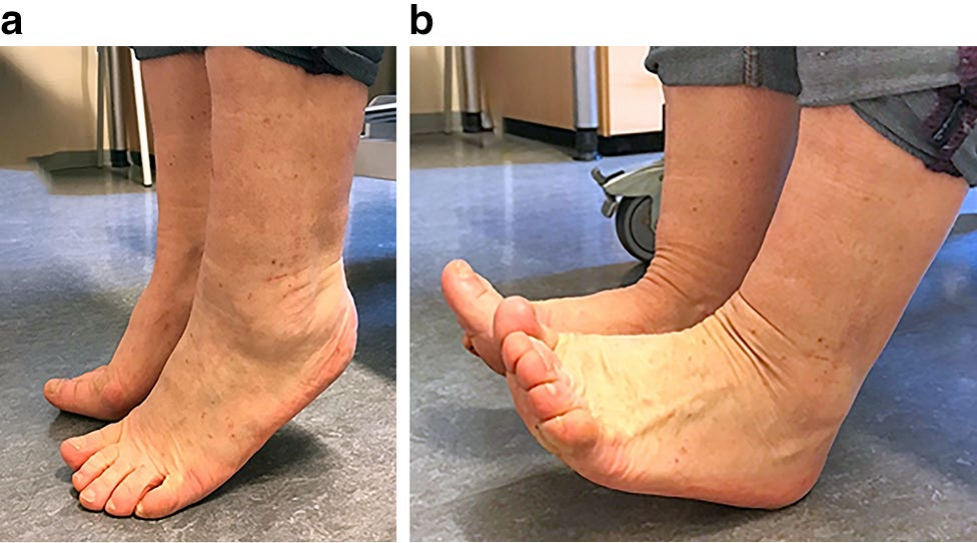
Free function (50 degrees of sagittal ankle motion on both sides) at 69-month follow-up (OMAS 100; AOFAS 100; FFI-D 1.85; SF-36 PCS 54.7 MCS 52.6) of the same patient shown in Figs. 1 and 4. Because the patient was pain free, no indication was seen for another set of radiographs at the time of follow-up

The sagittal range of motion of the surgically treated ankle joint averaged 44.4 (SD: 16.33) degrees. This was on average 3.4 (SD: 6.63) degrees (7%) less than that of the opposite side. In 77% of the patients there were no side-to-side differences in the range of motion (Fig. 4). All scores showed lower mean values with larger deficits in the range of motion measured at follow-up. The differences were not statistically significant for the OMAS (*p* = 0.101), FFI (*p* = 0.080); AOFAS (*p* = 0.053); SF-36 PCS (*p* = 0.163); or SF-36 MCS (*p* = 0.602).

Outcome scores in relation to fracture morphology are summarized in Table 1. There were similar values for range of motion, grade of osteoarthritis and outcome scores for bimalleolar, trimalleolar, and quadrimalleolar fracture patterns. No statistically significant correlation between the type of PM fracture and the results in any of the scores was seen. The presence of an intercalary fragment did not correlate with any of the scores, the grade of osteoarthritis, or the range of motion. Patients with PE-4 fractures displayed a significantly (*p* = 0.026) greater deficit in range of motion (average 5.53°; *SD: 6.85*) compared with PA-3 fractures (average 2.86°; *SD: 7.29*). Patients with an open fracture had a significantly lower FFI compared to those with closed fractures (46.9, *SD: 33.58* vs. 15.3, *SD: 20.25*; *p* = 0.024).

**Table 1:** Results vs. type of injury

Patient characteristics	*n* (%)	OMAS	FFI-D	AOFAS	SF-36 PCS	SF-36 MCS
Male	31	80.48 (SD:24.30)	17.88 (SD:25.34)	86.94 (SD:20.13)	48.48 (SD:12.93)	50.56 (SD:8.69)
Female	69	80.0 (SD:24.03)	15.82 (SD:19.69)	87.80 (SD:18.83)	47.37 (SD:12.40)	50.41 (SD:9.71)
*P* value		0.705	0.542	0.950	0.411	0.763
Type of fracture						
Bimalleolar fracture	12	78.33 (SD:26.05)	17.76 (SD:23.76)	85.25 (SD:19.50)	45.72 (SD:12.8)	50.94 (SD:10.83)
Trimalleolar fracture	57	79.65 (SD:25.16)	16.4 (SD:21.29)	86.54 (SD:21.24)	47.49 (SD:13.52)	49.92 (SD:9.69)
Quadrimalleolar fracture	31	81.77 (SD:21.55)	16.08 (SD:21.67)	90.23 (SD:14.7)	48.94 (SD:10.53)	51.29 (SD:8.29)
*P* value		0.743	0.899	0.579	0.861	0.969
Danis–Weber classification						
B	76	79.74 (SD:25.11)	16.02 (SD:21.21)	86.87 (SD:20.27)	47.62 (SD:12.51)	50.13 (SD:9.86)
C	24	81.46 (SD:20.46)	17.88 (SD:22.75)	89.62 (SD:15.22)	48.02 (SD:12.79)	51.47 (SD:7.69)
*P* value		0.889	0.648	0.654	0.935	0.624
Lauge–Hansen classification						
SE 3	4	71.25 (SD:34.49)	20.76 (SD:27.18)	75.25 (SD:27.54)	42.56 (SD:7.51)	46.9 (SD:3.7)
SE 4	26	75.38 (SD:24.16)	20.13 (SD:22.31)	85.69 (SD:19.47)	44.85 (SD:13.78)	48.46 (SD:10.54)
PA 3	46	82.93 (SD:24.89)	13.28 (SD:20.12)	88.54 (SD:20.2)	49.67 (SD:11.85)	51.39 (SD:9.13)
PE 4	24	81.46 (SD:20.46)	17.88 (SD:22.75)	89.62 (SD:15.22)	48.02 (SD:12.79)	51.47 (SD:7.69)
*P* value		0.470	0.461	0.318	0.289	0.344
Bartoníček-Rammelt classification						
Type 1	7	81.43 (SD:20.35)	11.31 (SD:12.96)	86.29 (SD:13.97)	44.92 (SD:16.57)	44.84 (SD:10.42)
Type 2	35	78.14 (SD:22.69)	18.71 (SD:21.31)	86.46 (SD:19.06)	46.36 (SD:12.60)	50.82 (SD:8.50)
Type 3	35	85.86 (SD:9.57)	13.12 (SD:20.39)	92.06 (SD:14.79)	49.51 (SD:10.48)	51.54 (SD:9.46)
Type 4	23	74.13 (SD:31.50)	19.71 (SD:25.34)	82.65 (SD:25.23)	47.97 (SD:14.22)	50.02 (SD:10.11)
*P* value		0.396	0.441	0.221	0.814	0.391
Intercalary fragment	33	88.58 (SD:17.62)	17.6 (SD:23.37)	81.67 (SD:24.46)	48.48 (SD:11.80)	51.73 (SD:8.87)
No intercalary fragment	67	87.01 (SD:19.96)	15.9 (SD:20.66)	79.4 (SD:24.46)	47.33 (SD:12.92)	49.82 (SD:9.60)
*P* value		0.629	0.965	0.526	0.635	0.472
Soft tissue damage						
Open fracture	4	62.5 (SD:31.75)	46.9 (SD:33.58)	67.0 (SD:38.54)	34.48 (SD:18.35)	48.9 (SD:10.37)
Closed fracture	96	81.17 (SD:23.23)	15.26 (SD:20.25)	88.56 (SD:17.7)	48.27 (SD:12.03)	50.52 (SD:9.37)
*P* value		0.118	**0.024**	0.062	0.137	0.814
Correlation coefficient, *r*		− 0.151	0.291	− 0.220	− 0.218	− 0.034

Significant difference value is printed in bold

Because there were 100 patients, the total number (*n*) per subgroup equals the percentage (%)

*OMAS* Olerud Molander Ankle Score, *FFI* Foot Function Index, *AOFAS* American Orthopedic Foot and Ankle Society Ankle/Hindfoot Score, *SF-36 PCS* physical health component summary scores of the Short Form Health 36, *SF-36 MCS* mental health component summary scores of the Short Form Health 36, *SE* supination external rotation fracture, *PA* pronation abduction fracture, *PE* pronation external rotation fracture

### Radiological results

A complete set of postoperative radiographs for detailed evaluation was available for 87 patients. In 63 cases (81.8%), no intra-articular step-off was seen. In 10 patients (13.0%), a step-off of 1–2 mm and in 4 cases (5.2%) a step-off of ≥ 2 mm was detected. Fibular shortening was seen in a single case (1.3%). At follow-up, 45 recent X-rays of the affected ankle were available. According to the Kellgren and Lawrence (KL) scale the average grade of osteoarthritis on the former fractured side was 1.8 (*SD: 0.96*). The opposite side showed an average KL grade of 0.37 (*SD: 0.54*).

Accuracy of reduction of the distal fibula into the tibial incisura was assessed in 13 postoperative CT scans (Fig. 2b). In 8 cases (61.5%) the position was judged as perfect. In 2 patients (15.4%) there was an anterior shift of the fibula compared to the opposite side. In 3 patients there was a posterior shift of the fibula (23.1%). In all cases, fibular shift was within less than 2 mm of the opposite side. Therefore, no revision surgery became necessary.

Correlation of clinical and radiological results is summarized in Table 2. There was a tendency towards inferior results with posterior fibular translation using the FFI (*p* = 0.066) and anterior translation of the fibula and the AOFAS score (*p* = 0.059). A persistent step-off in the articular surface of the tibia was associated with inferior outcome in all scores except SF-36 PCS. These differences did not reach statistical significance. Likewise, a difference of more than 2 KL grades of osteoarthritis negatively affected the results in all scores without reaching statistical significance.

**Table 2:** Results vs. radiographic parameters

Radiographic parameters	*n*	OMAS	FFI-D	AOFAS	SF-36 PCS	SF-36 MCS
Step-off						
< 1 mm	63	81.75 (SD:24.05)	14.54 (SD:20.67)	87.38 (20.94)	48,64 (SD:12,22)	50.56 (SD:9.34)
1–2 mm	10	77.5 (SD:22.52)	24.98 (SD:29.18)	87.50 (SD:11.02)	47.27 (SD:13.73)	51.14 (SD:7.62)
> 2 mm	4	60.00 (SD:38.08)	22.68 (SD:27.19)	77.25 (SD:26.30)	37,96 (SD:13,82)	44.94 (SD:12.94)
* P* value		0.442	0.621	0.402	0.261	0.551
Fibular position in the tibial incisura						
Correct position	8	80.63 (SD:26.79)	10.88 (SD:20.24)	88.00 (SD:22.53)	47.42 (SD:12.4)	45.81 (SD:11.72)
Too far anterior (1 mm)	2	42.50 (SD:31.82)	46.13 (SD:58.26)	46.50 (SD:44.55)	33.99 (SD:6.78)	43.59 (SD:2.43)
Too far posterior (1 mm)	3	66.67 (SD:41.63)	29.94 (SD:26.93)	78.00 (SD:38.11)	45.51 (SD:11.39)	45.67 (SD:4.40)
* P* value		0.293	0.137	0.174	0.364	0.461
Difference in KL osteoarthritis grade						
0	7	70.71 (SD:29.64)	30.21 (SD:31.89)	79.43 (SD:24.87)	45.69 (SD:4.29)	49.09 (SD:7.55)
1	17	76.76 (SD:23.71)	16.64 (SD:18.89)	88.29 (SD:13.32)	46.69 (SD:12.0)	48.85 (SD:11.06)
2	15	64.33 (SD:25.41)	27.99 (SD:16.91)	77.33 (SD:22.1)	39.79 (SD:13.06)	46.23 (SD:9.43)
3	1	35.0 (SD:0)	24.69 (SD:0)	60.0 (SD:0)	36.66 (SD:0)	60.52 (SD:0)
4	1	90 (SD:0)	13.58 (SD:0)	90.0 (SD:0)	36.97 (SD:0)	45.20 (SD:0)
*P* value		0.596	0.249	0.294	0.570	0.354

Because there were 100 patients, the total number (*n*) per subgroup equals the percentage (%)

*OMAS* Olerud Molander Ankle Score, *FFI* Foot Function Index, *AOFAS* American Orthopedic Foot & Ankle Society Ankle/Hindfoot Score, *SF-36 PCS* physical health component summary scores of the Short Form Health 36, *SF-36 MCS* mental health component summary scores of the Short Form Health 36, *KL* Kellgren–Lawrence

**Fig. 5:**
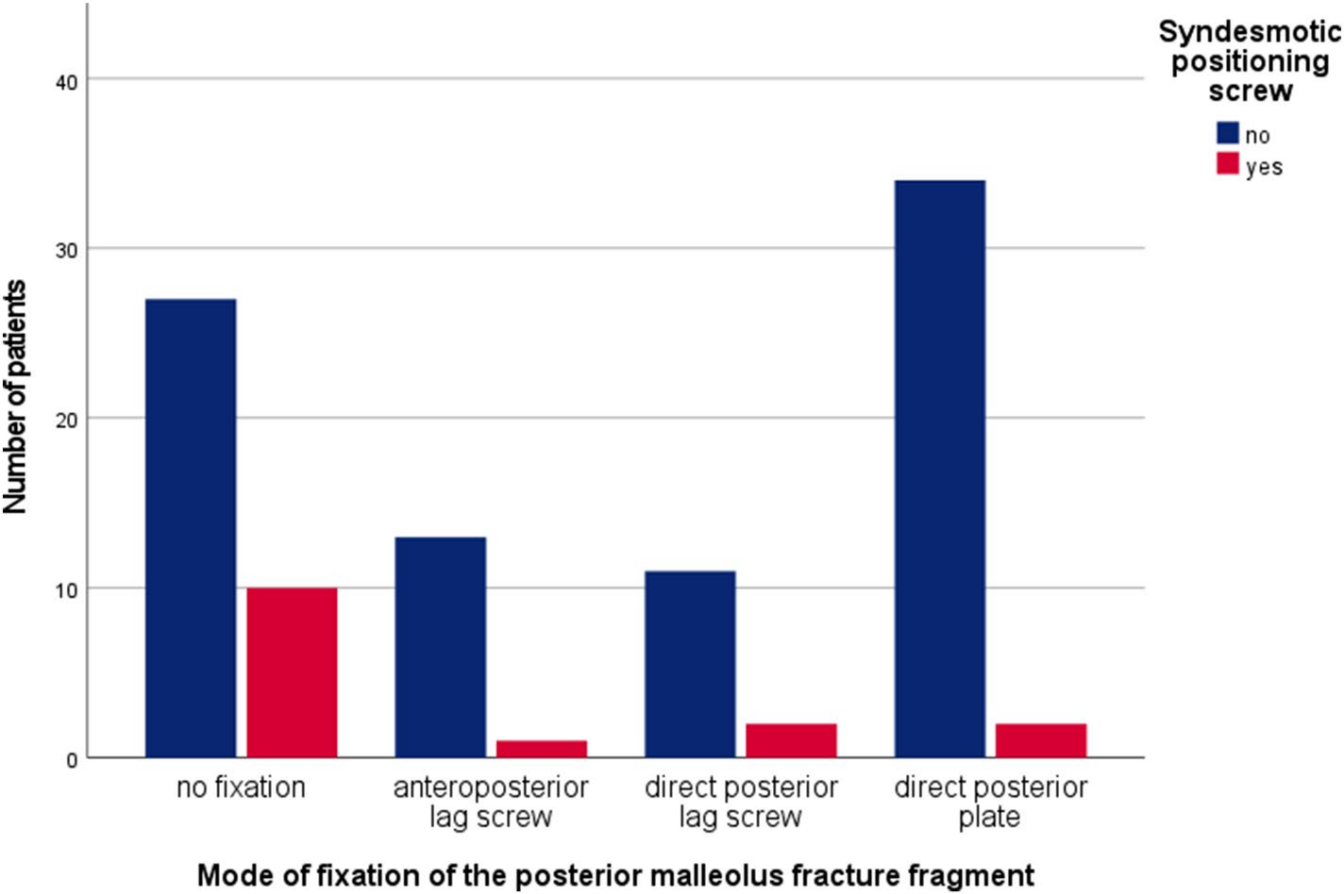
Mode of posterior malleolar fracture fixation in relation to the use of a syndesmotic positioning screw. There is a statistically significant correlation between no fixation of the posterior malleolus fracture fragment and the insertion of a syndesmotic positioning screw (*p* = 0.010)

Outcome scores in relation to the type of fracture fixation are summarized in Table 3. Patients who underwent primary internal fixation had significantly superior results with the SF-36 MCS than patients who underwent primary closed reduction and external fixation with staged internal fixation (*p* = 0.031). The type of definite internal fixation did not correlate with the presence of osteoarthritis, restricted mobility or the results in any of the scores. There was a statistically significant (*p* = 0.010) negative correlation between internal fixation of the PM fragment and the need for syndesmotic screw placement at the time of surgery (Fig. 5).

**Table 3:** Results vs. surgical treatment

Patient characteristics	*n*	OMAS	FFI-D	AOFAS	SF-36 PCS	SF-36 MCS
Primary surgical treatment						
Internal fixation		82.92 (SD:22.69)	14.09 (SD:19.61)	89.74 (SD:17.30)	48.25 (SD:12.17)	52.42 (SD:8.8)
External fixation		77.02 (SD:25.17)	19.14 (SD:23.35)	85.04 (SD:20.94)	47.12 (SD:12.99)	48.29 (SD:9.57)
* P* value		0.143	0.187	0.249	0.806	**0.031**
Fixation of posterior malleolar fragment						
No fixation	37	80.68 (SD:20.49)	15.34 (SD:18.87)	87.59 (SD:17.36)	46.63 (SD:12.75)	49.3 (SD:9.34)
AP screw	14	82.86 (SD:22.76)	11.04 (SD:14.58)	89.71 (SD:15.18)	49.73 (SD:11.3)	52.7 (SD:10.18)
PA screw	13	78.85 (SD:27.78)	18.97 (SD:27.30)	87.96 (SD:20.41)	50.7 (SD:11.46)	48.19 (SD:9.81)
Plate	36	79.03 (SD:27.09)	18.82 (SD:24.17)	86.56 (SD:22.25)	47.06 (SD:13.27)	51.54 (SD:8.96)
* P* value		0.812	0.572	0.874	0.660	0.241
Syndesmotic positioning screw	15	83.00 (SD:21.94)	14.96 (SD:20.20)	87.93 (SD:17.68)	49.01 (SD:13.00)	52.67 (SD:8.08)
No syndesmotic positioning screw	85	79.65 (SD:24.42)	16.73 (SD:21.81)	87.46 (SD:19.49)	47.48 (SD:12.49)	50.12 (SD:9.57)
* P* value		0.829	0.658	0.936	0.668	0.364
Implant removal						
Complete	50	82.10 (SD:23.13)	16.49 (SD:20.28)	88.28 (SD:18.27)	46.99 (SD:11.71)	51.28 (SD:10.53)
Partial	14	88.46 (SD:18.75)	11.65 (SD:24.86)	94.15 (SD:9.97)	54.77 (SD:7.73)	53.52 (SD:5.66)
No removal	36	75.42 (SD:25.87)	16.96 (SD:21.36)	84.47 (SD:22.44)	46.71 (SD:14.07)	47.84 (SD:8.05)
*P* value		0.091	0.437	0.217	0.089	**0.027**

Significant difference values are printed in bold

Because there were 100 patients, the total number (*n*) per subgroup equals the percentage (%)

*OMAS* Olerud Molander Ankle Score, *FFI* Foot Function Index, *AOFAS* American Orthopedic Foot & Ankle Society Ankle/Hindfoot Score, *SF-36 PCS* physical health component summary scores of the Short Form Health 36, *SF-36 MCS* mental health component summary scores of the Short Form Health 36, *AP-screw*anteroposterior lag screw, *PA-screw* posteroanterior lag screw

## Discussion

The aim of our retrospective study was to assess and present the injury characteristics and the medium-to-long-term clinical and functional outcome of surgical treatment of ankle fractures with a PM fragment in a larger patient population.

So far, only few studies on the surgical treatment of PM fractures have assessed outcome with general health questionnaires. Kang et al., in a Korean patient population, found a SF-36 of 91 at an average of 2 years following screw fixation for PM fractures [32]. Other studies on ankle fractures without an explicit mention of PM fragments also showed poorer long-term results in the SF-36 Score compared to the national population norm [32, 33].

Meijer et al. found a mean SF-36 PCS of 50 and MCS of 48 in a Dutch patient cohort at a mean follow-up of 24 years after PM fractures. In their study, larger fragments (> 25% of the articular surface in lateral radiographs) were treated with screw fixation. The authors concluded that psychological symptoms (depression, impairment) merit greater attention in the long-term after ankle fractures. However, they also found that better arc of plantarflexion/dorsiflexion and eversion/inversion correlated with better Foot and Ankle Outcome Scores and SF-36 PCS, respectively [34].

Our study is the first to report general health status on a larger patient cohort from Germany. In contrast to the study by Meijer et al. [34], we found a SF-36 PCS of 48 which was lower than the population norm and a SF-36 MCS of 50.5 that was even slightly higher than the population norm. Similar to Meijer et al. [34], we found superior scores with better range of motion; however, the differences did not reach statistical significance. This may be due to the fact, that 77% of the patients did not display any deficits in range of motion at final follow-up.

The average OMAS score of 80 in our study lies well within the range the scores reported in 4 studies over the last 10 years that range from 72 to 91 [16, 35–37]. The average AOFAS score of 88 in our study compares favorably with the results from the literature since 2004 which range from 69 to 96 [16, 32, 34–58].

Similar to Tuček et al. [38], we found a significant female preponderance for Bartoníček–Rammelt type 3 and 4 PM fractures. It may be speculated that particularly elderly women with osteoporotic bone quality are prone to more severe fracture patterns. However, a significant correlation between age and PM fracture type could not be demonstrated in both studies. Intercalary fragments were seen most frequently in Bartoníček–Rammelt type 2 and 3 fractures, which is in accordance to recent studies [20, 25].

Patients who underwent primary internal fixation had significantly superior SF-36 MCS and insignificantly higher scores with the other questionnaires than patients who underwent primary closed reduction and external fixation with staged internal fixation. This may reflect a more severe fracture pattern and soft tissue injury at the time of presentation as well as a longer hospitalization for the patients treated with staged internal fixation.

On the other hand, the method of definite internal fixation did not correlate with the presence of osteoarthritis, restricted mobility or the results in any of the scores. This may be explained by the fact that the fixation methods were tailored to the type of fracture. Like other authors [18, 59, 60] we found that internal fixation of the PM significantly reduced the need for syndesmotic screw placement at the time of surgery (*p* = 0.010). Fixation even of small, displaced PM fragments provides a physiologic, bone-to-bone fixation of the posterior tibiofibular ligament and thus increases syndesmotic stability [22, 61, 62]. Furthermore, direct fixation of bony avulsions provides a more physiologic means of syndesmotic stabilization than screws or flexible implants that have an inherent risk of malreduction and the need for secondary removal if symptomatic [63, 64].

In our practice, we employed an individualized approach to PM fractures according to the fracture pattern (see Fig. 2). We retrospectively included patients since 2003 for this study and the treatment has evolved over time. In general, Bartoníček–Rammelt type 1 fractures were treated non-operatively. Type 2 and 3 fractures, if displaced and / or impacted were treated with open reduction and direct posterior to anterior screw and plate fixation [22]. Type 4 (large triangular) PM fractures were treated with indirect anterior-to-posterior screw fixation if a transfibular visual control of reduction was possible [65]. Otherwise, direct fixation via a posterolateral approach was performed (see Fig. 3). With this treatment regimen, good overall results could be achieved throughout all fracture patterns. As pointed out by numerous authors, detailed preoperative analysis including CT imaging of the PM fracture is essential for the indication to surgery and planning the surgical approach [20, 22, 25, 62, 66–72]. In the present study, there were similar values for range of motion, grades of osteoarthritis and outcome scores for bimalleolar, trimalleolar and quadrimalleolar fracture patterns. No statistically significant correlation between the type of PM fracture or the presence of an intercalary fragment and the results in any of the scores, the grade of osteoarthritis, or the range of motion. This indicates an achievement, as traditionally, ankle fractures involving the PM were fraught with a less favorable prognosis [1, 5–9, 16, 47, 73–76].

In our study, we did not find a correlation between the size of the PM fragment and functional or radiographic outcome. This is in line with several other clinical studies [40, 50, 73, 74, 77], while others have found inferior functional results and higher degrees of osteoarthritis with increasing fragment size [1, 16, 19, 47, 75, 78]. It appears from the existing data that fragment size is one of the prognostic factors as it may to some extent reflect the fracture energy, but by far not the only one [22].

Several clinical studies have identified a postoperative step-off of 1–2 mm as an independent risk factor for inferior outcome and the development of posttraumatic ankle osteoarthritis irrespective of the size if the PM fragment [47, 55, 62, 73, 77, 79]. In our study, the presence of a joint step-off of > 1 mm resulted in insignificantly inferior scores, probably due to the low number of patients with residual steps. The latter also did not correlate with the development of osteoarthritis. The prevalence of osteoarthritis in our study may have been overestimated, as follow-up radiographs were obtained in symptomatic patients only. We conclude from our results and those from the literature that anatomic joint reconstruction is essential for a good clinical outcome after PM fractures.

In the presence of syndesmotic disruption, correct positioning of the fibula within the tibial incisura is crucial for obtaining a favorable outcome after malleolar fractures [22, 64, 80–84]. Similarly, we found a trend to inferior outcome scores even with slight (1 mm) anterior or posterior shift of the fibula after internal fixation. The differences did not reach significance because of the small amount of displacement and low overall numbers (only 2 patients with anterior shift and 3 patients with posterior shift of the fibula). Incorrect positioning of the fibula significantly alterates the distribution of forces within the ankle joint which, in the long term, may lead to posttraumatic arthritis [85, 86].

Our study has several limitations. These include the retrospective study design and loss to follow-up of more than two thirds of the eligible patients. This lies within the range of 32–75% loss to follow up in similar medium to long-term studies on this subject [34, 40, 73, 74, 77, 82]. In the present study, there was a combination of a relatively old patient age, a long follow-up period, and the large catchment area of our center. On the other hand, a substantial number of patients were seen in person at a mean follow-up of 7 years, while many studies use questionnaires or phone interviews. Furthermore, at the time of initial management, we did not routinely employ CT imaging for patients with PM fractures. Therefore, for 45% of patients, only plain X-ray images were available to assess the pathoanatomy of the PM fragment. It may be speculated, that several fractures could have been underestimated in our study, because important features like intercalary fragments, medial fracture extension, incisura involvement and impaction of the joint surface cannot reliably be detected without CT scanning [70]. The same is true for postoperative CT scans to assess correct anatomical reduction the fibula into the tibial incisura. Finally, we used the non-validated AOFAS score as one of our outcome measurements, because it has been the most commonly used score and allows some comparison with other studies. However, it was employed along with several validated scores including patient-reported outcome measurements.

In conclusion, with anatomic reduction and stable internal fixation tailored to the individual pathoanatomy, the mere presence of a PM fragment does not lead to a poor outcome in ankle fractures. The size of the PM fragment alone is not of prognostic relevance. Exact reconstruction of the articular surface including impaction of the plafond, restoration of the tibial incisura and tibiofibular alignment as well as syndesmotic stability are prerequisites for favorable treatment results. Preoperative analysis and assessment of the fracture pattern with CT imaging is important for individual treatment planning. Fixation of PM fragments significantly reduces the need for additional tibiofibular fixation.
